# Beliefs about cannabis at the time of legalization in Canada: results from a general population survey

**DOI:** 10.1186/s12954-019-0353-z

**Published:** 2020-01-06

**Authors:** John A. Cunningham

**Affiliations:** 1 Centre for Addiction and Mental Health, 33 Russell St, Toronto, Ontario M5S 2S1 Canada; 2 University of Toronto, Toronto, Canada; 3 Australian National University, Canberra, Australia

**Keywords:** Cannabis, Legalization, Epidemiology, Societal beliefs

## Abstract

**Aims:**

Cannabis became a legally available drug in Canada in October of 2018. The objective of this study was to examine beliefs about cannabis use at the time of legalization among past year cannabis users, those who had used cannabis but not in the last year, and people who had never used cannabis.

**Design and methods:**

A survey of 813 participants, 18 years and over, and identified using random digit dialing methods, was made of the Canadian general population. Among other items, participants were asked a series of questions about their beliefs regarding cannabis use and recovery.

**Results:**

Compared to never and ever users, participants who used cannabis in the last year regarded cannabis as less of a societal problem (mean [SD] past year use = 3.8 [2.4] versus 6.4 [2.6] and 6.0 [2.4] respectively, *p* = .001), than people were less likely to become addicted to cannabis if they tried it (past year use = 13.3% versus 48.3% and 25.1%, *p* = .001), and a larger proportion believed that recovery from cannabis without treatment was likely (past year use = 40.8% versus 14.2% and 19.3%, *p* = .001). All groups were equally positive of the chances of recovering from cannabis addiction with treatment (*p* = .72).

**Discussion and conclusion:**

Beliefs about cannabis use vary substantially between those who have used the drug in the past year and those who have not. Replication of the survey at a later date is merited in order to assess the ways in which beliefs about cannabis evolve after an extended period of cannabis being available as a legal drug.

After many decades being classified as an illegal drug in Canada, cannabis had its status changed to that of a legal drug in 2018. While cannabis has been viewed by the general public as a relatively benign drug for some time [1–5], it is possible that recent social movements, political activity around the build-up to legalization, as well as movements towards legalization in some states of the USA, have modified attitudes even further [6–8]. Such information may be important for the health of Canadians, as well as the provision of health services, because societal views of an activity or a substance can influence health-related behaviors (such as the use of cannabis) as well as the provision of services (such as addictions treatment) [9].

In 2018, a general population telephone survey was conducted in Canada as part of a larger project examining perceptions of drug use [10]. This survey incorporated a series of questions assessing societal attitudes towards cannabis use, as well participants’ own experience with the drug (and other drugs). Beliefs about cannabis use were compared between participants who had used cannabis (ever and past year) to those who had never used cannabis. Further, in order to capture potential changes in attitudes to cannabis around the time of legalization, half of the participants were assessed prior to when cannabis became legally available in October of 2018 (interviews conducted in August of 2018) and the other half were assessed immediately after (November 2018). Comparisons between the two 2018 samples were made to assess any changes in attitudes and beliefs immediately around legalization.

## Methods

The current survey was a replication of one conducted in 2008. Details of the survey development and sampling strategy for the 2008 survey are provided elsewhere [1, 2]. Briefly, the survey comprised of a random digit dialing sample of Canadian households, including both landline and cell phone samples, and provided in both English and French. The study received ethics approval from the research ethics board of the Centre for Addiction and Mental Health. As this was a telephone survey, participants provided verbal consent to participate prior to commencing the study. Independent samples were generated for survey components conducted after cannabis became legally available. The project was described to potential participants as a survey about people’s beliefs regarding the use of alcohol, tobacco, and other drugs. Participants were selected from households by asking to speak to the person 18 years or older with the next birthday. Briefly, among other items, participants were asked a series of questions assessing (1) how serious they thought cannabis was as a societal problem (1 = not at all serious; 10 = very serious; 6.4% of participants did not reply to this question); (2) the risk of developing cannabis addiction if you try the drug (8% missing data; 1 = no risk, 5 = very high risk; re-categorized as 4 or 5 = high risk, 1–3 = low risk); (3) the chances of people fixing cannabis addiction on their own (without treatment) (6.8% missing data; response categories: 1 = no chance, 6 = very large chance; re-categorized as 5 or 6 = high chance, 1–4 = low chance for these analyses); and (4) the chance of fixing cannabis addiction if the person went to treatment (9.5% missing data; response categories: 1 = no chance, 6 = very large chance; re-categorized as 5 or 6 = high chance, 1–4 = low chance for these analyses). Finally, participants were asked about their own use of cannabis (never, prior to past year, in the past 12 months; 1.3% missing data).

### Analysis plan

After examining the distribution of variables and recoding as necessary, bivariate comparisons of opinions about cannabis use were made between those who responded prior to and after the date cannabis was legally available. Analyses were then conducted to explore variations in opinions between the different cannabis consumption groups (never used, prior to past year use, and past year use) employing one-way ANOVA and chi-square analyses. Analyses are reported based on weighted values. Sample sizes are reported as unweighted samples.

## Results

The landline sample had a response rate of 30% and the cell phone response rate was 10%, leading to a weighted average overall response rate of 23%. Demographic characteristics are presented for the whole sample (*N* = 813) between those who had never used cannabis (*n* = 491), ever used (i.e., prior to past year use; *n* = 225), or used cannabis in the last year (*n* = 97; see Table 1). Participants who reported using cannabis in the last year were younger than those who had never or ever used (mean [SD] past year use = 38.7 [16.3] versus 51.8 [18.9] and 49.7 [14.1] respectively; *F* = 27.8, *p* =.001; Scheffé post hoc test). Participants who had ever used cannabis appeared more likely to report some post-secondary education (79.4%) compared to those who had never used (69.1%) or used in the past year (68.8; *χ*^2^ = 8.4, 2 df, *p* = .015). Finally, people reporting ever using or past year cannabis use appeared to be more likely to report being full or part time employed (69.6% and 74.0% respectively) compared to those who reported never having used cannabis (53.2%; *χ*^2^ = 27.2, 2 df, *p* = .001).

**Table 1:** Demographic characteristics by cannabis use status

	Cannabis use status			*p*
	Never used (*n* = 491)	Ever used (*n* = 225)	Past year use (*n* = 97)	
Mean (SD) age	51.8 (18.9)	49.7 (19.2)	38.7 (16.3)	.001
% Male	49.9	44.5	52.8	.27
% Married/common law	65.6	64.8	57.3	.22
% Some post-secondary	69.1	79.4	68.8	.015
% Household income < $30,000	10.5	11.6	11.8	.90
% Full/part time employed	53.2	69.6	74.0	.001

There were no significant differences (*p* > .05) in participants’ opinions about cannabis before and after it was legally available for sale in Canada (i.e., before and after October 1, 2018). As such, Table 2 displays results that combine the two time period samples and comparisons are presented between the three cannabis use categories. Participants who had used cannabis in the last year were more likely to view cannabis as only a minor societal problem compared to those who had never or ever used cannabis (mean [SD] past year use = 3.8 [2.4] versus 6.4 [2.6] and 6.0 [2.4] respectively; *F* = 51.9, 2 df, *p* = .001; Scheffé post hoc test). Similarly, past year cannabis users were unlikely to believe that there was a large chance of developing an addiction to cannabis after trying the drug (13.3%), whereas about half of those who never used thought addiction was a large possibility (48.3%) and a quarter of ever users believed in this possibility (25.1%; *χ*^2^ = 65.3, 2 df, *p* = .001). About 40% of past year users thought those who were addicted to cannabis could fix the addiction without treatment (40.8%) while belief in this possibility was much lower among never and ever users (14.2% and 19.3%; *χ*^2^ = 42.2, 2 df, *p* = .001). Finally, there was little difference in the cannabis use groups in their belief that there was a large chance of fixing cannabis addiction through treatment with about two-thirds in each group rating a large possibility of fixing a cannabis addiction with treatment (*p* = .72).

**Table 2:** Comparing opinions about cannabis between never, ever, and past year cannabis users

	Never (*n* = 491)	Ever (*n* = 225)	Past year (*n* = 97)	*p*
Mean (SD) seriousness of Cannabis use as a societal problem^a^	6.4 (2.6)	6.0 (2.4)	3.8 (2.4)	.001
% High risk of addiction if try	48.3	25.1	13.3	.001
% Large chance of fixing addiction on own, without treatment	14.2	19.3	40.8	.001
% Large chance of fixing addiction with treatment	63.4	64.4	67.8	.72

^a^1 = not at all serious; 10 = extremely serious

## Discussion

The legalization of cannabis in Canada was a significant shift in drug control policy that reflected a shift in views about the dangers associated with cannabis use. Around the time that legalization came into force, the general public (as measured in this survey) appeared to view cannabis as of relatively low risk. Unsurprisingly, past year cannabis users regarded cannabis as a less serious problem with a lower risk potential than prior to past year cannabis users and those who reported never trying the drug. While the chances of fixing a cannabis addiction without treatment were regarded as fairly low (and about which past year cannabis users were more confident than the other participants), all participants appeared fairly confident that cannabis addiction could be fixed with treatment.

In recent years, a number of other nationally (or state) representative surveys of attitudes to cannabis have been conducted in the USA [5, 7, 8]. Similar to this Canadian survey, cannabis use was generally regarded as low risk in these samples. However, direct comparisons of questions are not possible between these surveys due to the different questions used in each project.

There appeared to be little difference in opinions about cannabis use immediately before and after cannabis was legally available (analyses not shown as none approached statistical significance at *p* > .05). Perhaps this time period was too limited to examine any changes in beliefs about cannabis and a repetition of the survey in a year (or several years’ time) might find an evolution of beliefs about cannabis resulting from an increase in availability. Not anticipated by the author was the fact that cannabis, while legally available after October of 2018, was not actually readily commercially available in many regions of Canada (due to delays in implementing commercial retail options at the provincial level and an inadequate production and distribution network). This lack of availability could have played a role in the finding that beliefs did not appear to change in the months before and after legal availability.

Other limitations of the current project included a low response rate to the survey. While low response rates are common, they do lead to concerns regarding the representativeness of the sample. In addition, it would have been valuable to have a more detailed assessment of level and severity of cannabis use among past year consumers. However, for this information to have been useful, the sample size for the survey would have needed to have been substantially larger in order to recruit a sufficient number of regular cannabis users to conduct the analyses.

### Conclusion

The results demonstrate the substantial differences in opinions about cannabis use between those who have never used and those with some experience with the drug. It will be interesting to see how these opinions change as legal availability of cannabis increases over time, the prevalence of cannabis use (most probably) also increases, and patterns of cannabis-related harm are modified.

## Data Availability

Available from the corresponding author on reasonable request.
